# Supplementary material for: Efficient thermal face recognition method using optimized curvelet features for biometric authentication
Source: PLoS One. 2023 Jun 26;18(6):e0287349. doi: 10.1371/journal.pone.0287349 (PMC10292695; doi:10.1371/journal.pone.0287349)
Supplement: Attachment — Submitted filename: response_to_Editor PLOS One.pdf [file pone.0287349.s002.pdf]

Response to Editor

Article ID: PONE-D-23-08936

Original Article Title: Efficient thermal face recognition method using optimized curvelet features for biometric authentication

Re: Response to Editor

Dear Editor-in-chief,

Thank you for allowing a resubmission of our manuscript, with an opportunity to address the reviewers' comments. We appreciate the Editor and the reviewers for taking the time to review our manuscript carefully and for providing valuable feedback to help us improve the quality of the document.
We have made every effort to eliminate all the indicated major and minor inconsistencies and do hope that as a result, the quality of our revised manuscript has improved further. The updated parts are highlighted in red color.

Point 1: The readability and presentation of the study should be further improved. The paper suffers from language problems

Response 1: We appreciate this comment. We restructured and improved the flow of the paragraphs of the paper to improve its readability and presentation. We also carefully proofread the entire paper

---

.

Point 2: "Discussion" section should be edited in a more highlighting, argumentative way. The author should analysis the reason why the tested results is achieved. It will be helpful to the readers if some discussions about insight of the main results are added as Remarks.

Response 2: Thanks for pointing out this important issue. We added 3 subsections in the discussion. Section, 7.1 Research Questions Discussion which discusses the results in terms of two research questions in an argumentative way and identifies Remarks from this discussion. In Section, 7.2, we discussed the possible applications of the proposed models while in Section 7.3, we compared and discussed our model with the most related work

---

Point 3: How to set the parameters of proposed method for better performance?

Response 3: The parameter's best values were identified in presented in Tables 2-7.

Point 4: The significance of the design carried out in this paper is not well explained relative to other important works published in this field. The authors should review, comment, and compare more works that are developed recently. The authors should clearly emphasize the contribution of the study. Please note that the up-to-date of references will contribute to the up-to-date of your manuscript. The study named " Recognition of COVID-19 disease from X-ray images by hybrid model consisting of 2D curvelet transform, chaotic salp swarm algorithm and deep learning technique; Artificial intelligence-based robust hybrid algorithm design and implementation for real-time detection of plant diseases in agricultural environments"- can be used to explain the object detection process in the study.

Response 4: Thank you so much for your valuable comment. Responding to this comment, we did the following:

1- cited the recommended articles, See lines 40-40
2- added the significance, the challenge and the gap in knowledge of the study in the introduction section, See lines 64-83
3- The contribution of the study has been re-stated and emphasized, See lines 79-118

Point 5: The main contributions of the study can be given in the last paragraph of the Introduction section.

Response 5: Thank you so much for your valuable comment.

The contribution of the study has been re-stated and emphasized, See lines 79-118
